# Evaluation of the hinotori^™^ Surgical Robot System for accurate suturing in small cavities

**DOI:** 10.1007/s11701-024-02053-y

**Published:** 2024-07-27

**Authors:** Yasuyuki Kameoka, Yuichi Okata, Shohei Yoshimura, Shino Inuzuka, Serena Iwabuchi, Harunori Miyauchi, Taichi Nakatani, Yuichiro Tomioka, Yuki Takanarita, Yuko Bitoh

**Affiliations:** 1 Division of Pediatric Surgery, Department of Surgery, Kobe University Graduate School of Medicine, Postal Address: 7-5-2, Kusunoki-cho, Chuo-ku, Kobe, Hyogo 650-0017 Japan; 2 Department of Pediatric Surgery, Hyogo Prefectural Kobe Children’s Hospital, Kobe, Japan; 3 Clinical and Translational Research Center, Kobe University Hospital, Kobe, Japan; 4 Department of Pediatric Surgery, Hyogo Prefectural Harima-Himeji General Medical Center, Himeji, Japan

**Keywords:** Pediatric surgery, Robotics, Laparoscopy, Computer-assisted laparoscopy, Hinotori, Preclinical study

## Abstract

The hinotori^™^ Surgical Robot System (hinotori^™^, Medicaroid, Kobe, Japan) is increasingly being utilized primarily in urology and adult surgery; however, data on its application in pediatric surgery are lacking. This preclinical study aimed to evaluate the limitations of this system for accurate suturing in small cavities designed for pediatric and neonatal applications. Two trained operators performed simple ligature sutures (easy task [ET]) and hepaticojejunostomy sutures (difficult task [DT]) within five differently sized boxes, ranging from 5123 to 125 mL. The suture time, number of internal and external instrument/instrument collisions, instrument/box collisions, and suture accuracy were evaluated. The suture accuracy was assessed using the A-Lap Mini endoscopic surgery skill assessment system. As a result, an increase in the number of collisions and extended suturing times were observed in boxes with volumes smaller than 215 mL. Despite these variations, there were no significant differences between the boxes, and all tasks were precisely performed in all boxes (*p* = 0.10 for the ET and *p* = 1.00 for the DT). These findings demonstrate the capability of the hinotori^™^ system to perform precise suturing techniques within tightly confined simulated neonatal cavities as small as 125 mL. To advance the integration of pediatric robotic surgery utilizing the hinotori^™^ system, additional trials comparing it with conventional laparoscopic and thoracoscopic techniques using pediatric and animal models are necessary to assess its clinical safety and applicability.

## Introduction

The safety and efficacy of robot-assisted surgery have been established in the pediatric field, with its use steadily expanding to various pediatric surgical subspecialties [1–3]. Additionally, continuous innovations in robotic technology, including the development of new platforms, miniaturization of instruments, and cost reduction, are expected to accelerate the adoption of pediatric robotic surgeries [2, 4, 5].

The hinotori^™^ Surgical Robot System (hereafter referred to as the hinotori™ system), introduced in 2019 by Medicaroid Inc. in Kobe, Japan, is a novel robot-assisted surgical platform with several notable features. Each robotic arm has eight axes of movement and a slim profile compared to da Vinci’s seven axes, which helps to minimize collisions. Moreover, its docking-free design autonomously calibrates trocar positions (pivot positions), increasing the space around the trocars and reducing the risk of abdominal wall tissue damage owing to excessive traction. An integrated alert function prevents arm interference from outside the body cavity, thereby enhancing procedural safety. Additionally, a flexible three-dimensional (3D) viewer in the surgeon’s cockpit may alleviate neck and shoulder fatigue. The hinotori^™^ system is primarily utilized in urology, adult surgery, and gynecology [6–15]. While there are currently no reports of its use in pediatric surgery, it is anticipated that hinotori™ will expand to this field in the future.

However, in pediatric robot-assisted surgery, the minimum body size for a safe operation has not yet been clearly defined. Previous studies have demonstrated the feasibility of performing surgical procedures in small spaces designed for neonates using the da Vinci Surgical System (da Vinci^™^, Intuitive Surgical, United States) [16, 17], Versius^®^ Surgical Robotic System (Versius^®^, CMR Surgical, United Kingdom) [18], and Senhance Robotic Surgical System (Senhance^™^, TransEnterix, Italy) [19]. Previous research has indicated that suturing tasks can be successfully executed within confined spaces with minimum volumes ranging from 90 to 125 mL. However, the spatial constraints within which the hinotori^™^ system can safely perform surgical procedures have not been empirically defined. Consequently, there is a notable lack of data on the technical feasibility of employing the hinotori^™^ system in such restricted environments.

Therefore, in this study, we aimed to assess the limitations of accurate suturing in small spaces designed for pediatric and neonatal applications using the hinotori^™^ system.

## Materials and methods

### Study setting and equipment

**Fig. 1:**
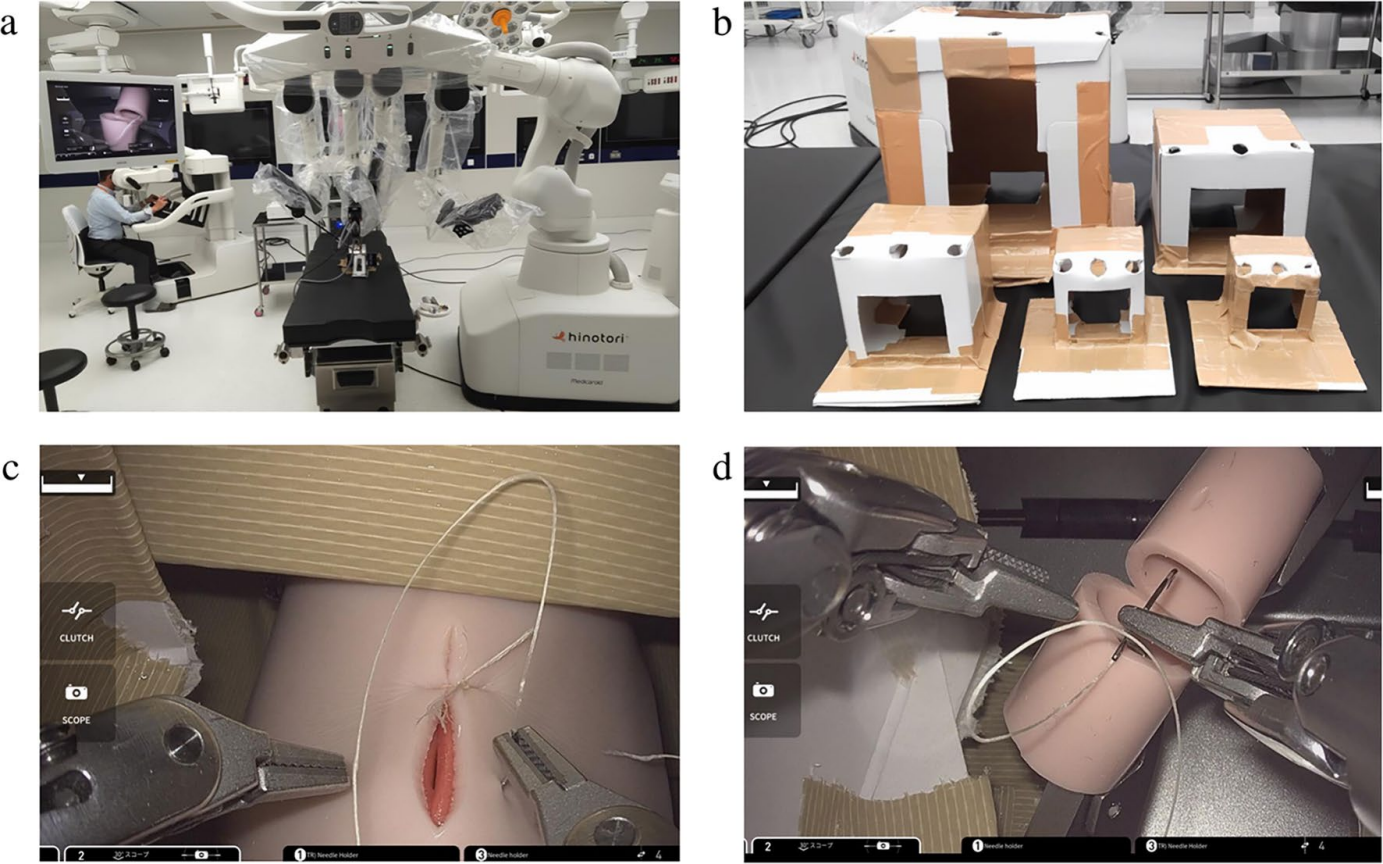
**a** Verification setup. The box is secured to the stand, and the suturing inside the box is recorded simultaneously using a mobile phone camera and a monitor. **b** The five cardboard boxes with decreasing volume. A trapdoor is cut into the anterior and posterior surfaces to record the procedure and place the suturing device. The ports are arranged in a straight line as shown in the diagram, and the suturing target is positioned on the opposite side of the camera port within the box. **c** The easy task of suturing with A-Lap Mini inside the box. **d** The difficult task of suturing the posterior wall of the intestine positioned 6 mm within the lumen, a procedure akin to pediatric laparoscopic hepaticojejunostomy

This study was conducted at Medicaroid Corporation facilities using the hinotori^™^ system. The experimental setup consisted of a master console and three robotic arms, each equipped with a 10 mm 30° videoscope and 2 5 mm needle holders (Fig. 1a).

Five custom-made cardboard boxes were used to simulate pediatric cavities and were modified to incorporate cutouts for suturing targets and external observations; height, length, width, distance between ports, and volume were recorded (Table 1). These boxes, reinforced with duct tape for added structural integrity, were secured to a standard operating table (SOT-100 Vercia^™^, Medicaroid, Japan) with duct tape and configured with two 8 mm ports and 10 mm camera ports in a linear arrangement (Fig. 1b).

**Table 1:** Size of the cardboard boxes, the calculated volume

Box no	Box height (cm)	Box length (cm)	Box width (cm)	Box volume(ml)	ΔLC (cm)	ΔRC (cm)
1	18	18	18	5832	8.5	8.5
2	12	12	10	1440	5.5	5.5
3	8	8	8	512	3.5	3.5
4	6	6	6	216	2.5	2.5
5	5	5	5	125	2	2

ΔLC (cm) distance of the left instrument to the camera, ΔRC (cm) distance of the right instrument to the camera

### Participants and consent

In this study, two certified trainers from the Medicaroid Corporation, each with more than 3 years of experience in handling the hinotori^™^ system, were selected as operators. These trainers had achieved a high level of proficiency with the hinotori^™^ system, having completed extensive training and practice that allowed them to perform suturing tasks efficiently. Given their experience and prior certification, they had surpassed the learning curve necessary for the operations performed in this study.

The ethics committee at our institution determined that this study did not require an institutional review board approval. Informed consent was obtained from the trainers for the participation, evaluation, and publication of the study results.

### Surgical tasks and protocol

The study involved two distinct suturing tasks designed to evaluate the safety and performance limits within small cavities. The easy task (ET) involved three single-knot sutures on a simulated intestine using the A-Lap Mini Endoscopic Surgery Skill Assessment System, which includes a model of an artificial intestine designed to mimic four tissue layers: mucosal, submucosal, muscle, and serosal (A-Lap Mini, Kyoto Kagaku Co. Ltd, Kyoto, Japan). The difficult task (DT) required three sutures for the anastomosis of the posterior wall of the intestine positioned 6 mm within the lumen, a procedure akin to pediatric laparoscopic hepaticojejunostomy (Fig. 1c, d). The operators were not pre-informed of the tasks, and no practice period was set. The tasks were performed in five custom-made cardboard boxes with dimensions recorded as width × height × length, facilitating the evaluation under various spatial constraints. The camera port and target were placed on the opposite sides of each box, and suturing was executed using 5–0 Vicryl sutures with TF-1 needles (Ethicon, Jonson, and Jonson, Neuss, Germany). All surgical procedures were video-recorded for subsequent blinded evaluations.

Each operator sequentially performed the ET and DT once in each box, starting with the largest box. After completion, the box was replaced with progressively smaller boxes (from 18 × 18 × 18 cm to 5 × 5 × 5 cm) [16–19]. All boxes used in this study were designed to simulate the volume of the abdominal cavity in infants and neonates under 1 year: Box 1 (5832 ml) for a 1 year-old, 10 kg; Box 2 (1440 ml) for a 6 month-old, 8 kg; Box 3 (512 ml) for a 3 month-old, 6 kg; Box 4 (216 ml) for a 1 month-old, 4 kg; and Box 5 (125 ml) for a neonate, 3 kg [20, 21].

The performance precision was assessed using the A-Lap Mini Endoscopic Surgery Skill Assessment System. This is an objective and quantitative assessment system that examines the performance of suture ligature methods in an artificial intestinal anastomosis model that utilizes 4 layers: mucosal, submucosal, muscle, and serosal. These intestinal suture sheets simulate human tissue, enabling quantitative assessments of performance time, air pressure leak volume, suture tension, number of full-thickness sutures, and wound opening area [22–24]. Each parameter received a score of up to 5 points, with a maximum total score of 25 points. This scoring system, designed to reflect the performance standards of experienced surgeons, has been widely adopted in several publications to objectively evaluate the accuracy of surgical techniques. After each task, the operators did not receive feedback on the A-Lap Mini scores to avoid influencing their performance. For the DT, we employed a simulated hepaticojejunostomy model using a portable surgical training tool from the FUKUSHIN Surgeon’s Tool Laboratory (Hyogo, Japan). The suturing accuracy was scored by evaluators who checked the suture bite width, the presence of any slack in the knots, and the presence of any damage to the simulated intestinal tract. Each suture was scored 1 point if all stitches were correctly placed, and the maximum achievable score was 3 points, which serves as a quantitative measure of suturing accuracy [25].

### Blinded evaluation and performance assessment

The recorded procedures were evaluated by an independent third party who was blinded to the identity of the operators and the specifics of the experimental setup.

### Outcome measures and statistical analysis

The outcome measures for this study included the suturing time (s), which encompasses needle handling and knot tying times, internal and external instrument/instrument collisions (alert numbers), and instrument/box collisions. The suturing accuracy was assessed using the A-Lap Mini score for the ET and a 3-point scale for the DT. Importantly, the hinotori^™^ system is equipped with an alert function designed specifically to prevent collisions between the robotic arms and external obstacles, such as the sides of the surgical box. This function provides auditory warnings and automatically stops the arms to avoid potential collisions. However, it is crucial to note that this alert system does not manage or prevent collisions between forceps inside the box.

To facilitate the comparison, data on suturing time and collision incidents were plotted against decreasing box volume. The medians of each dataset were compared using the Kruskal–Wallis test conducted using EZR (64-bit) analysis software [26]. Statistical significance was set at *p* < 0.05.

## Results

Two operators were able to complete the ET and DT in all boxes prepared in advance, with volumes ranging from 5860 to 125 mL.

In the ET, no significant differences were observed in the overall procedure time required to complete the tasks (*p* = 0.11). However, as the box volume decreased to 216 mL or less, a trend toward longer knot-tying times was noted, with the suture time divided into needle handling and knot-tying components (*p* = 0.72 and *p* = 0.11, respectively) (Table 2). Similarly, in the DT, although there were no variations in the task procedure and needle handling time (*p* = 0.23 and *p* = 0.99, respectively), the knot-tying time increased when the box volume was similarly reduced, consistent with the findings in the ET (*p* = 0.13) (Table 3).

**Table 2:** Median suture time, collision, and the A-Lap Mini score per box for the easy task

Box no	1 (5832 ml)	2 (1440 ml)	3 (512 ml)	4 (216 ml)	5 (125 ml)	*p-*value
Time (s)						
Suture	369.0 [350–388]	294.0 [276–312]	337.5 [289–386]	491.0 [477–505]	540.0 [429–651]	0.111
Needle handling	96.5 [96–97]	67.0 [54–80]	87.5 [64–111]	79.0 [58–100]	114.5 [64–165]	0.715
Knot tying	272.5 [253–293]	227.0 [222–232]	250.0 [225–275]	412.0 [405–419]	425.5 [365–486]	0.111
Collision (n)						
Internal instrument/instrument	11.5 [11–12]	4.0 [3–5]	5.5 [4–7]	42.5 [6–15]	10.0 [6–14]	0.280
External instrument/instrument ※ alert numbers	0.0 [0–0]	0.0 [0–0]	5.5 [1–10]	42.5 [38–47]	50.0 [30–70]	0.079
Internal instrument/box	0.0 [0–0]	0.0 [0–0]	1.5 [0–3]	5.5 [3–8]	2.5 [1–4]	0.148
Suture Accuracy (score)						
A Lap-Mini total Score	12.0 [12–12]	18.0 [17–19]	13.0 [10–16]	15.5 [14–17]	19.0 [18–20]	0.125
Performance time	3.0 [3–3]	4.0 [4–4]	3.5 [3–4]	2.0 [2–2]	2.5 [2–3]	
Volume of air pressure leak	0.0 [0–0]	1.0 [1–1]	0.0 [0–0]	0.5 [0–1]	3.0 [1–5]	
Suture tension	2.0 [2–2]	4.5 [4–5]	2.5 [2–3]	3.0 [2–4]	3.5 [3–4]	
Number of full-thickness sutures	2.0 [2–2]	3.5 [2–5]	3.5 [2–5]	5.0 [5–5]	5.0 [5–5]	
Wound opening area	5.0 [5–5]	5.0 [5–5]	3.5 [5–5]	5.0 [5–5]	5.0 [5–5]	

External instrument/instrument collision presents the total number of alerts

**Table 3:** Median suture time, collision, and the A-Lap Mini score per box for the difficult task

Box no	1 (5832 ml)	2 (1440 ml)	3 (512 ml)	4 (216 ml)	5 (125 ml)	*p*-value
Time (s)						
Suture	405.5 [395–416]	522.0 [405–369]	513.5 [472–555]	699.0 [623–775]	694.0 [587–801]	0.225
Needle handling	158.5 [138–179]	153.5 [112–195]	151.0 [86–130]	156.0 [130–182]	172.5 [130–215]	0.996
Knot tying	247.0 [216–278]	368.5 [293–444]	362.5 [339–386]	543.0 [441–645]	521.5 [457–586]	0.131
Collision (n)						
Internal instrument/instrument	4.0 [1–7]	6.50 [5–88]	15.5 [10–21]	40.0 [37–43]	35.0 [30–40]	0.089
External instrument/instrument ※ alert numbers	0.0 [0–0]	0.0 [0–0]	4.0 [3–5]	58.0 [54–62]	50.5 [44–57]	0.076
Internal instrument/box	0.0 [0–0]	0.0 [0–0]	4.0 [1–7]	5.0 [4–6]	6.5 [4–9]	0.125
Suture Accuracy (score)						
Total score	3 [3–3]	3 [3–3]	3 [3–3]	3 [3–3]	3 [3–3]	1.00

External instrument/instrument collision presents the total number of alerts

No significant differences were observed in internal, external, and instrument-box collisions in the ET (*p* = 0.28, *p* = 0.08, and *p* = 0.15, respectively) (Table 2) or DT (*p* = 0.10, *p* = 0.08, and *p* = 0.13, respectively) (Table 3). However, there was a noticeable trend indicating an increase in internal and external collisions when the box volume was reduced to 216 mL or less.

**Fig. 2:**
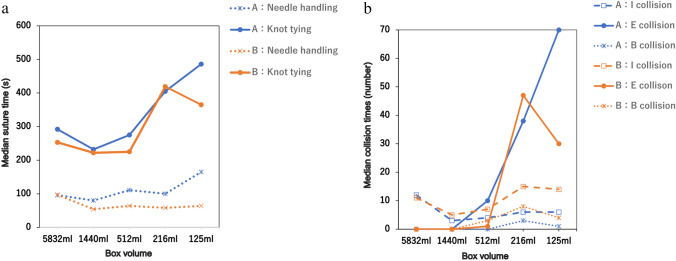
Easy task. **a** Boxplot of the mean suturing time (y-axis) is plotted against the boxes beginning in the largest box with descending size (x-axis). **b** Boxplot of the mean collision times (y-axis) is plotted against the boxes beginning in the largest box with descending size (x-axis). I collision: Internal instrument/instrument collision. E collision: External instrument/instrument collision (alert numbers). B collision: instrument/box collision. The two surgeons who possessed an extensive experience with the hinotori™ system are represented using different colors on the graph: one using an orange line and the other using a blue line

**Fig. 3:**
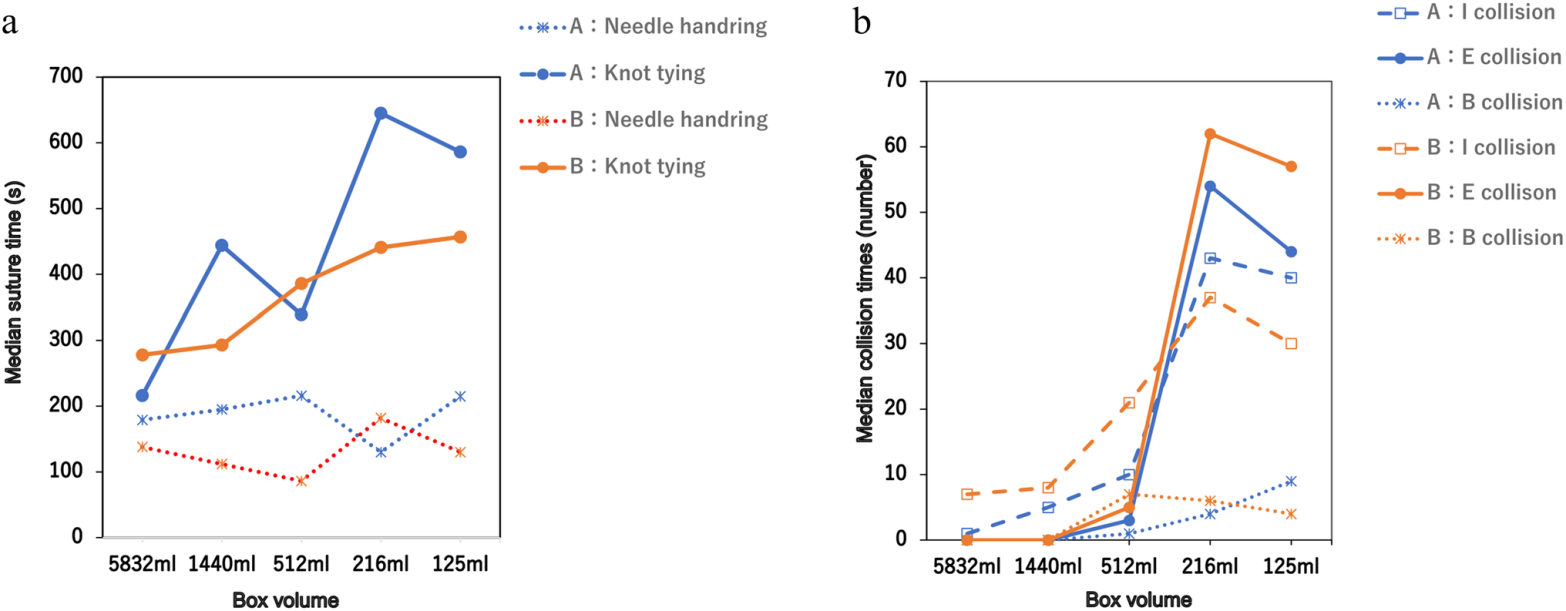
Difficult task. **a** Boxplot of the mean suturing time (y-axis) is plotted against the boxes beginning in the largest box with descending size (x-axis). **b** Boxplot of the mean collision times (y-axis) is plotted against the boxes beginning in the largest box with descending size (x-axis). I collision: Internal instrument/instrument collision. E collision: External instrument/instrument collision (alert numbers). B collision: instrument/box collision. The two surgeons who possessed an extensive experience with the hinotori™ system are represented using different colors on the graph: one using an orange line and the other using a blue line

Based on these results, the suture time and collision incidence were plotted against the decreasing box volume. These findings suggest a correlation between extended knot-tying times and increased instrument collisions when the box volume is reduced to 216 mL or less (Figs. 2a, b, 3a, b).

The suturing accuracy for the ET remained consistently high across all box sizes (*p* = 0.10) (Table 2). One of the scoring criteria for the A-Lap Mini includes time; when the completion of the ET took longer and resulted in lower scores as the box volume decreased, the overall A-Lap Mini score was the highest. In the DT, accurate suturing was achieved in all boxes (*p* = 1.00) (Table 3).

After all of the verifications, the boxes used showed no damage or deformation, and the ports remained intact without any cracks.

## Discussion

This is the first study to objectively evaluate suturing and its accuracy in small cavities designed for pediatric and neonatal applications using the hinotori™ system. Our results demonstrate that the hinotori™ system could complete each task while ensuring the accuracy of suturing within an operative cavity as small as 125 mL, with no external collisions. Although a number of alerts were initially classified as external collisions, no direct interference was observed owing to the effective control mechanism of the hinotori^™^ system.

A notable finding of this study was the increase in knot-tying times as the cavity size decreased to 216 mL or smaller, suggesting that spatial constraints significantly affect surgical dynamics. Despite the uniform box sizes, the DT showed a higher incidence of collisions than the ET did, indicating that complexity escalates in constrained environments. This is in line with previous findings, which suggest that tighter spaces augment the likelihood of procedural collisions [16–18]. Addressing these spatial challenges in future iterations of robotic systems could enhance the efficiency and safety of the procedure.

Interestingly, the needle handling times remained consistent across all box sizes, underscoring the robust performance of the system even under varying spatial conditions. This consistency is crucial for operations, such as tissue dissection, which require precise manipulation. The performance of the hinotori^™^ system suggests that it could provide significant advantages over traditional laparoscopic or open surgical approaches, especially in the confined spaces that are typical of pediatric surgeries.

Although previous studies have shown that robotic systems can operate effectively in confined spaces as small as 90–125 mL, our findings extend this knowledge by demonstrating efficient suturing in a 125 mL space using more complex tasks [16–19]. We also introduced nuanced assessments of suturing precision, which are critical for evaluating the capabilities of surgical robots in real-world scenarios. Despite not testing the smallest reported volume of 90 mL, the performance of the system with more demanding tasks suggests that it is on par with or superior to existing technologies.

One of the hinotori^™^ system’s distinguishing features is its ability to operate without fixed docking, a capability shared by systems like Versius^®^ and Senhance^™^. This allows for a minimal setup with port distances as short as 2 cm, which may enable “port-free” operations.

A key attribute of the hinotori^™^ system is its alert function, which is designed to halt operations to prevent contact between external instruments until corrective maneuvers are executed. This feature potentially increases the procedure time but contributes significantly to the safety of surgical operations, making it particularly valuable in neonatal surgeries where precision and caution are paramount.

Our study results indicate that the smallest (125 mL) box yielded the highest suturing accuracy scores, highlighting the system’s capability to maintain precision even in significantly constrained spaces. This finding is notable, especially considering that increased suturing times were observed, likely because of the frequent activation of the alert function and the resulting instrument collisions.

Moreover, the advantages of robotic surgery, such as enhanced visual recognition and magnification, play a crucial role in enabling precise suturing within small operational volumes. These technological benefits are critical, particularly when the surgical targets are consistent, supporting high precision in environments where traditional approaches may be challenging or infeasible.

The alert function of the hinotori*™* system significantly enhances surgical safety, which is particularly crucial in neonatal surgeries where precision is paramount. This feature, which is designed to halt operations and prevent contact with external instruments, supports surgical adjustments to prevent unintended contact. Although beneficial, this mechanism may extend the procedure times due to its sensitivity, which prompts frequent halts, especially when operating within the tightly constrained 125 mL space where the system demonstrated its highest suturing accuracy.

This study highlights that despite the limited operational space, the hinotori*™* system maintained excellent precision, demonstrating its capability to effectively manage surgical challenges associated with small cavities. The negligible learning curve associated with this system underscores its user-friendliness and efficiency, allowing surgeons to perform delicate tasks with enhanced visual recognition and magnification. These technological advantages are particularly beneficial in neonatal surgeries, where precision is critical, and the surgical area is inherently small.

Overall, the findings illustrate that the hinotori*™* system, while possibly slowing down procedures due to its alert function, offers significant advantages in terms of safety and accuracy in pediatric surgeries. The ability to perform accurately in restricted spaces without compromising safety demonstrates its potential as a valuable tool in complex surgical settings.

In pediatric robotic surgery, Meehan et al. reported that surgeries on children weighing less than 5 kg can generally be performed without significant issues, though the challenges increase markedly in cases involving children weighing less than 3 kg [27, 28]. Chapman et al. estimated that the volume of the right lung ranges from 92 to 140 mL in infants weighing 5 kg and from 55 to 84 mL in those weighing 3 kg [29]. Regarding the volume of the abdominal cavity, if we assume that the abdominal cavity of a neonate undergoing pneumoperitoneum for laparoscopy is spherical, the estimated volume for a neonate weighing 3 kg is approximately 125 mL [20]. These findings suggest that performing thoracoscopic surgery with the hinotori™ system might be particularly challenging in infants and neonates under 3 kg. In contrast, abdominal procedures might be more feasible at a younger age than thoracoscopic surgeries, owing to the softer abdominal walls of children compared with those of adults and the increased volume of the abdominal cavity owing to the pneumoperitoneum. This distinction is crucial in planning and executing surgical interventions using robotic systems in young patients.

### Limitations

Despite providing valuable insights, this study has some limitations. First, the generalizability of the results is constrained by the small sample size involving only two highly skilled operators, which may not accurately reflect the learning curve for less experienced surgeons. Additionally, we cannot deny the possibility that the operators' familiarity with the two tasks influenced the results. Furthermore, the simulation of pediatric surgical environments using custom-made cardboard boxes fails to replicate the complex and variable anatomy of real pediatric intracorporeal spaces, particularly the soft and elastic properties of neonatal abdominal walls. Moreover, the experimental setup did not mimic the dynamic operating conditions typically encountered during surgery, such as variations in lighting or patient movement, which could affect the surgical outcomes. Importantly, we did not include a comparative analysis with other robotic systems such as Versius^®^ and Senhance^™^, which is a critical aspect that should be addressed in future studies.

Given the limitations identified in this study, future research should aim to enhance the realism of surgical simulations using anatomically accurate 3D-printed models that closely mimic the physical properties of pediatric and neonatal tissues. Expanding the participant pool to include surgeons with varying levels of experience will provide a more comprehensive understanding of the learning curve associated with the hinotori^™^ system. Additionally, it would be beneficial to replicate these experiments in a more dynamic surgical environment to evaluate the system’s performance under typical operating room conditions, including factors such as lighting variations and patient movement. Moreover, future studies should include comparative analyses with other robotic surgical systems, such as Versius^®^ and Senhance^™^, to better assess the relative advantages and limitations of the hinotori^™^ system. These studies would not only validate the findings of the current research but may also broaden the applicability of the hinotori^™^ system in pediatric surgery, ensuring its effectiveness and safety in real-world clinical settings.

## Conclusion

This study demonstrated the capability of the hinotori^™^ Surgical Robot System to perform precise suturing techniques within tightly confined simulated neonatal cavities as small as 125 mL. This study substantiates the effectiveness of the system in managing complex suturing tasks under significantly restricted spatial conditions, with a level of accuracy that aligns with that of conventional surgical methods. To advance the integration of pediatric robotic surgery utilizing the hinotori^™^ system, it is imperative to undertake further comparative trials. These studies should involve traditional laparoscopic and thoracoscopic techniques, utilizing pediatric models and animal experiments to rigorously evaluate and verify the clinical applicability and safety of the system in realistic surgical environments.

## Data Availability

The data used to support the findings of this study are available from the corresponding author upon reasonable request.
